# Supplementary material for: DeepPHiC: predicting promoter-centered chromatin interactions using a novel deep learning approach
Source: Bioinformatics. 2022 Dec 10;39(1):btac801. doi: 10.1093/bioinformatics/btac801 (PMC9825766; doi:10.1093/bioinformatics/btac801)
Supplement: btac801_Supplementary_Data [file btac801_supplementary_data.pdf]

# Supplementary materials for DeepPHiC: Predicting promoter-centered chromatin interactions using a novel deep learning approach

## 1 Supplementary tables

| Tissue/Cell Type | Tissue/Cell Type Class | Abbr. | #PE (FDR<0.1) | #PP (FDR<0.1) |
|---|---|---|---|---|
| Adrenal gland | Primary Tissue | AD2 | 2556 | 5421 |
| Aorta | Primary Tissue | AO | 5224 | 788 |
| Bladder | Primary Tissue | BL1 | 4918 | 555 |
| Cardiomyocytes | Primary Tissue | CM | 7332 | 962 |
| Esophagus | Primary Tissue | EG2 | 20049 | - |
| Fat | Primary Tissue | FT2 | 56057 | - |
| Gastric tissue | Primary Tissue | GA | 1610 | 900 |
| GM12878+GM19240 Lymphoblastoid Cell Line | Primary Cell Line | GM | 4903 | 4446 |
| H1 Embryonic Stem Cell | Embryonic Stem Cell | H1 | 8178 | 917 |
| Hippocampus | Primary Tissue | HCmerge | 17434 | 2519 |
| Fibroblast cells | Primary Cell Line | IMR90 | 13217 | 4156 |
| Lung | Primary Tissue | LG | 1188 | 661 |
| Liver | Primary Tissue | LI11 | 5325 | 706 |
| Left Ventricle | Primary Tissue | LV | 1565 | 968 |
| H1-derived Mesendoderm Cell | Early Embryonic Lineages | ME | 1045 | 3142 |
| H1-derived Mesenchymal Stem Cell | Early Embryonic Lineages | MSC | 16379 | 4168 |
| H1-derived Neuronal Progenitor Cell | Early Embryonic Lineages | NPC | 3010 | 546 |
| Ovary | Primary Tissue | OV2 | 1105 | 187 |
| Pancreas | Primary Tissue | PA | 2594 | 396 |
| Psoas | Primary Tissue | PO3 | 1964 | 314 |
| Right Ventricle | Primary Tissue | RV | 1405 | 723 |
| Small Bowel | Primary Tissue | SB | 972 | 704 |
| Sigmoid colon | Primary Tissue | SG1 | - | 10338 |
| Spleen | Primary Tissue | SX | 22392 | 5866 |
| Trophoblast | Early Embryonic Lineages | TB | 83604 | 444 |
| Thymus | Primary Tissue | TH1 | 8993 | 1066 |
| Dorsorateral prefrontal cortex | Primary Tissue | X5628FC | 26084 | 6224 |

Table S1: Summary of promoter-enhancer and promoter-promoter interactions in 26 tissues/cell types

| Tissue/Cell Type | #PE | | #PP | |
|---|---|---|---|---|
| | Range | Median | Range | Median |
| Primary Tissue | 972 - 56057 | 3756 | 187 - 10338 | 788 |
| Primary Cell Line | 4903 - 13217 | 7332 | 962 - 4446 | 4156 |
| Embryonic Stem Cell | 8178 | 8178 | 917 | 917 |
| Early Embryonic Lineages | 1045 - 83604 | 9695 | 444 - 4168 | 1844 |

Table S2: Summary of promoter-enhancer and promoter-promoter interactions in four categories of tissues/cell types

| Tissue/Region | Roadmap Epigenomics (Chromatin Accessibility/Histone Modification |
|---|---|
| Adrenal gland | Pancreas Chromatin Accessibility |
| Aorta | Aorta H3K4me1 |
| Bladder | Fetal Kidney Chromatin Accessibility |
| Cardiomyocytes | Aorta H3K4me1 |
| Esophagus | Esophagus H3K4me1 |
| Fat | Adipose Tissue H3K27ac |
| Gastric tissue | Gastric Chromatin Accessibility |
| GM12878+GM19240 Lymphoblastoid Cell Line | Fetal Spleen Chromatin Accessibility |
| H1 Embryonic Stem Cell | H1 H3K4me3 |
| Hippocampus | Brain Hippocampus Middle H3K4me1 |
| Fibroblast cells | Breast Fibroblast Primary Cells H3K4me1 |
| Lung | Fetal Lung Right Chromatin Accessibility |
| Liver | Adult Liver H3K4me3 |
| Left Ventricle | Left Ventricle H3K4me1 |
| H1-derived Mesendoderm Cell | H1 Derived Mesenchymal Stem Cells H3K4me1 |
| H1-derived Mesenchymal Stem Cell | H1 Derived Mesenchymal Stem Cells H3K4me1 |
| H1-derived Neuronal Progenitor Cell | H1 Derived Neuronal Progenitor Cultured Cells H3K4me1 |
| Ovary | Fetal Ovary Chromatin Accessibility |
| Pancreas | Pancreas Chromatin Accessibility |
| Psoas | Psoas Muscle Chromatin Accessibility |
| Right Ventricle | Right Ventricle H3K4me1 |
| Small Bowel | Small Intestine Chromatin Accessibility |
| Sigmoid colon | Sigmoid Colon H3K4me1 |
| Spleen | Spleen H3K4me1 |
| Trophoblast | H1 BMP4 Derived Trophoblast Cultured Cells H3K4me1 |
| Thymus | Fetal Thymus Chromatin Accessibility |
| Dorsorateral prefrontal cortex | Fetal Brain Chromatin Accessibility |

Table S3: Summary of matched tissue/cell type-specific epigenetic data for pHi-C data

# 2 Supplementary figures

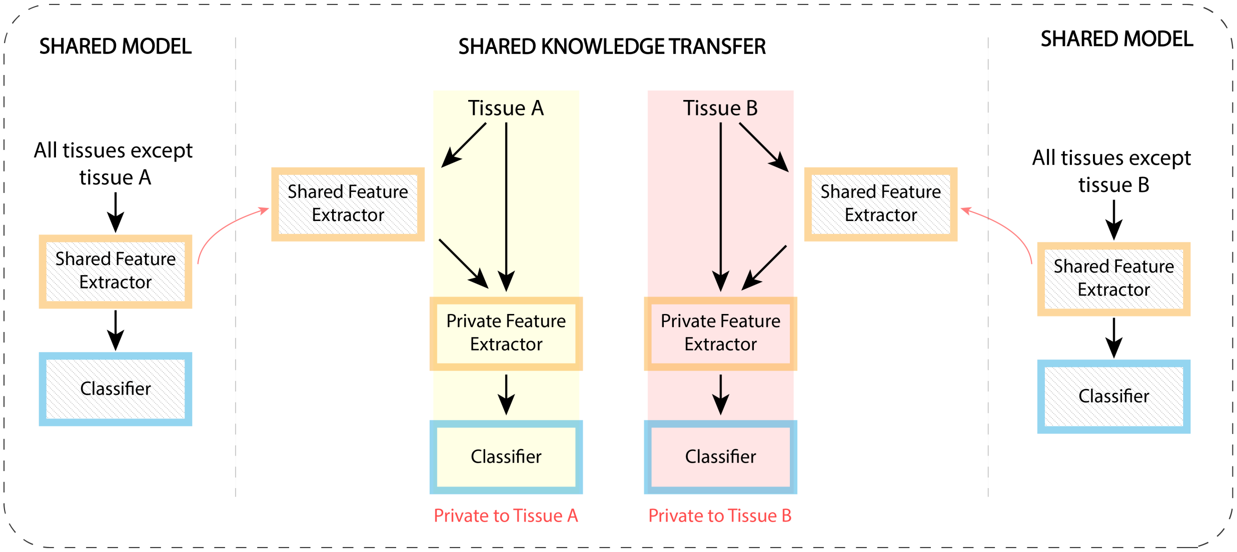


Figure S1: "Shared knowledge transfer" strategy for training he multi-task learning model, where we learn the shared feature extractor using all tissues except the tissue of interest (A or B) and transfer the shared feature extractor to a specific task for predicting chromatin interactions of tissue of interest (A or B).

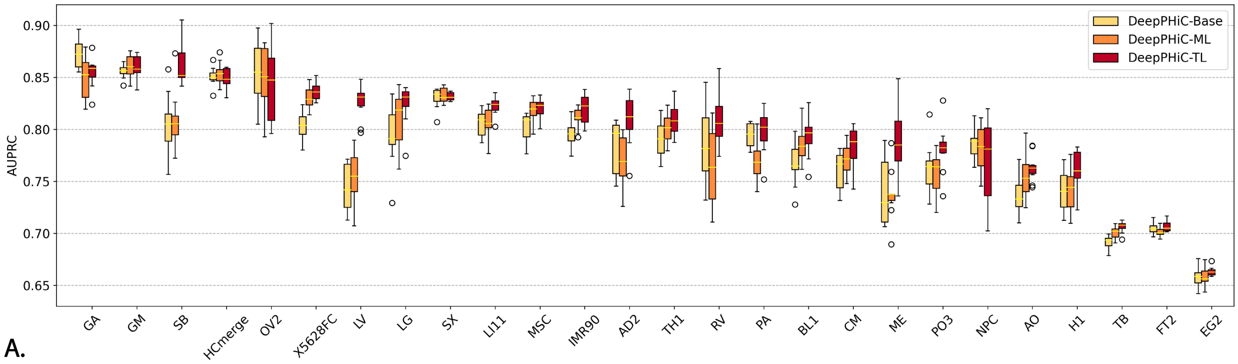


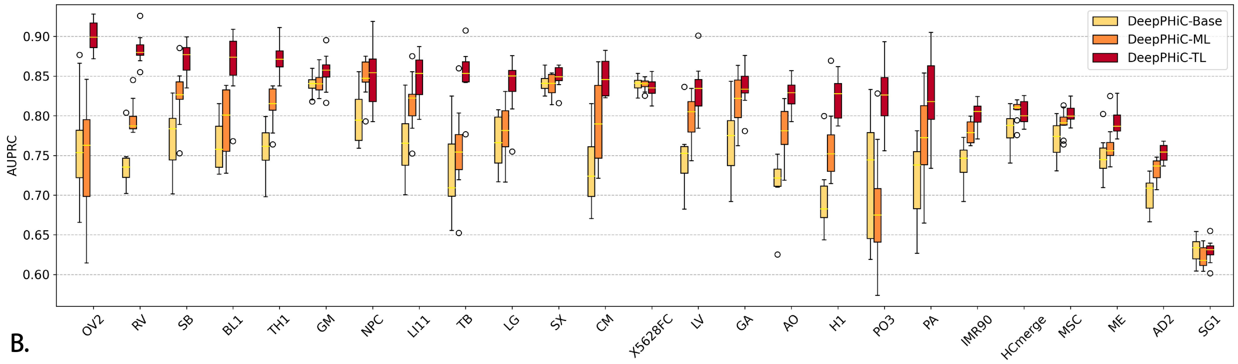


Figure S2: Comparison of three versions of DeepPHiC. **A.** promoter-enhancer interactions (PE) in 26 tissues/cell types and **B.** promoter-promoter interactions (PP) in 25 tissues/cell types. AUPRC are reported on 10 different experiments.

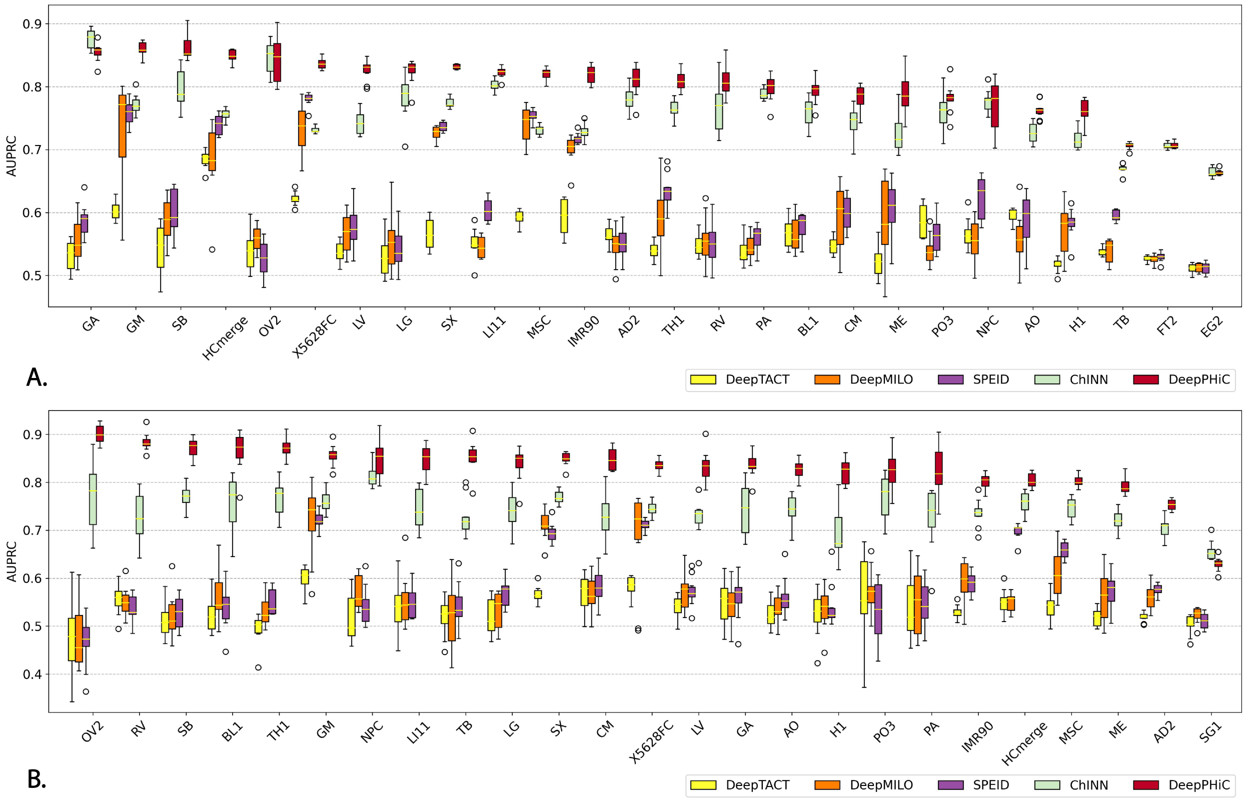


Figure S3: Comparison of DeepPHiC and state-of-the-art deep learning models on predicting **A.** Promoter-enhancer interactions (PE) and **B.** Promoter-promoter interactions (PP). Each boxplot represents the AUPRC in 10 experiments for each tissue.

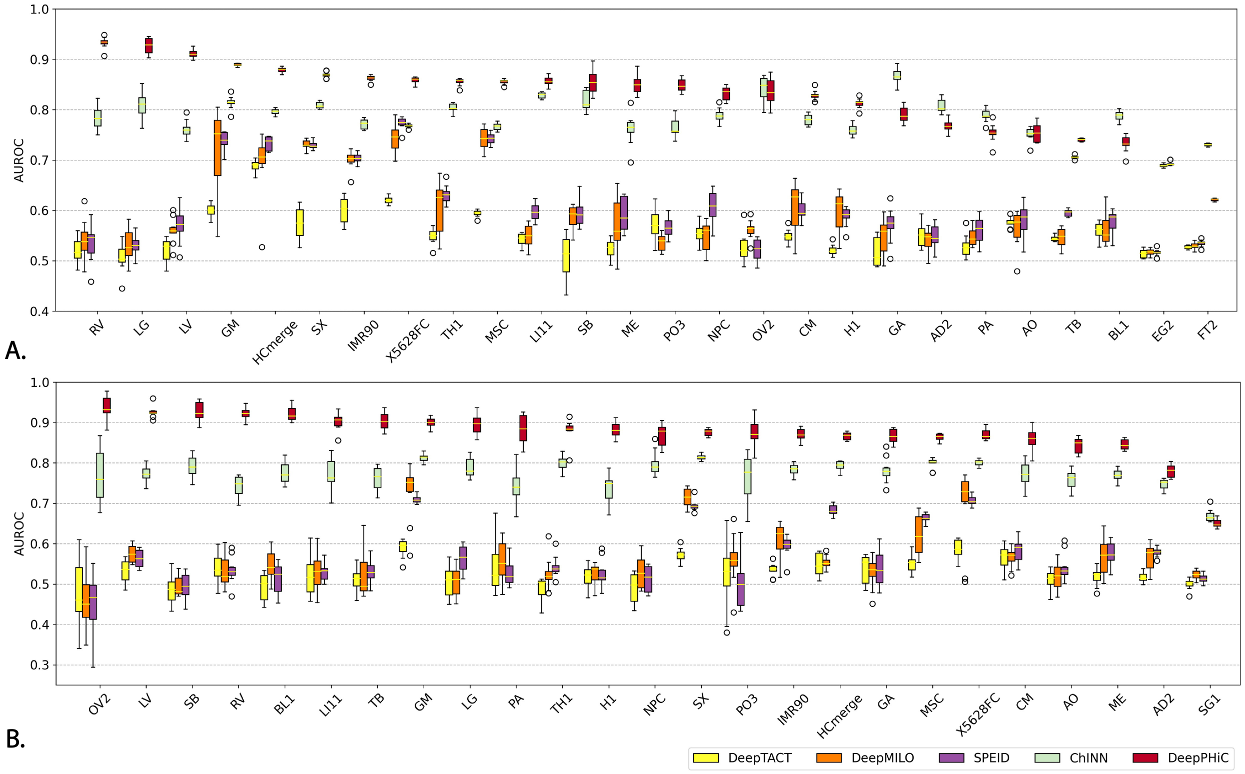


Figure S4: Comparison of DeepPHiC and state-of-the-art deep learning models on predicting **A.** Promoter-enhancer interactions (PE) and **B.** Promoter-promoter interactions (PP). Each boxplot represents the AUC in 10 experiments for each tissue. FDR cutoffs for constructing both positive set and negative set are FDR<0.05 and FDR>0.5.

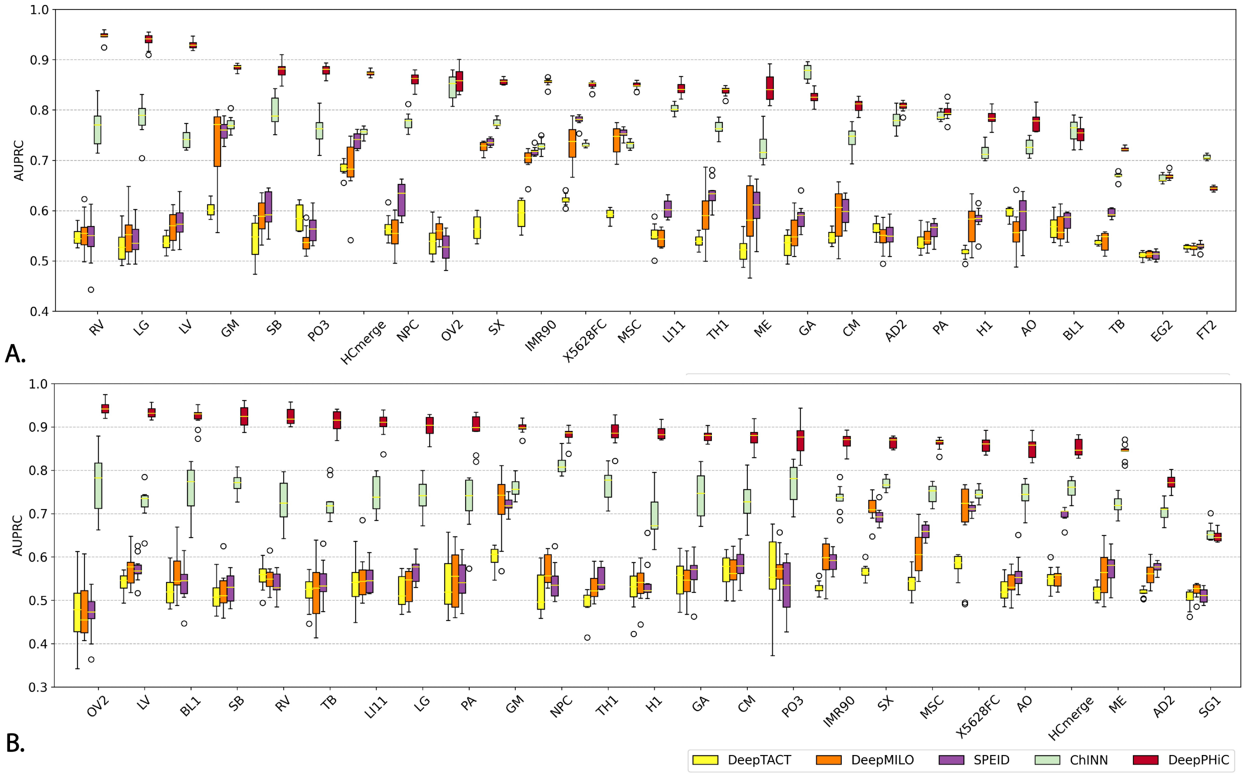


Figure S5: Comparison of DeepPHiC and state-of-the-art deep learning models on predicting **A.** Promoter-enhancer interactions (PE) and **B.** Promoter-promoter interactions (PP). Each boxplot represents the AUPRC in 10 experiments for each tissue. FDR cutoffs for constructing both positive set and negative set are FDR<0.05 and FDR>0.5.

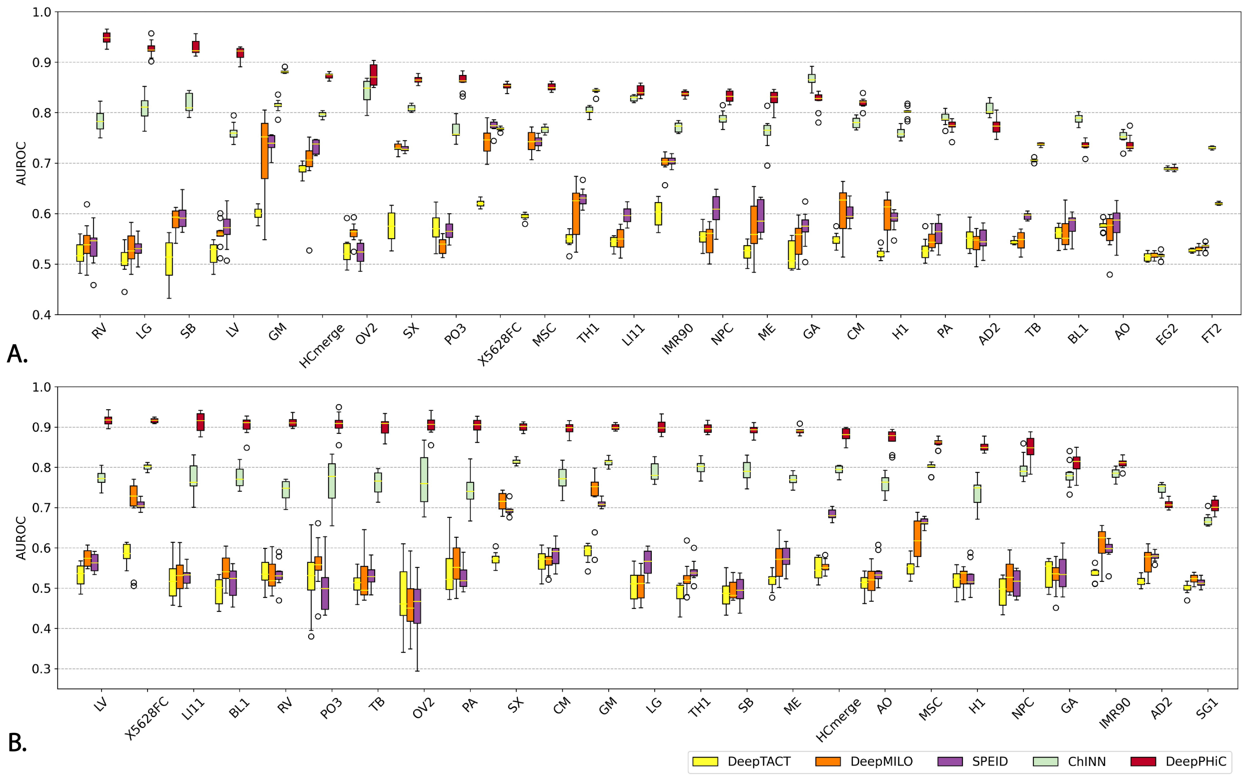


Figure S6: Comparison of DeepPHiC and state-of-the-art deep learning models on predicting **A.** Promoter-enhancer interactions (PE) and **B.** Promoter-promoter interactions (PP). Each boxplot represents the AUC in 10 experiments for each tissue. FDR cutoffs for constructing both positive set and negative set are FDR<0.1 and FDR>0.7.

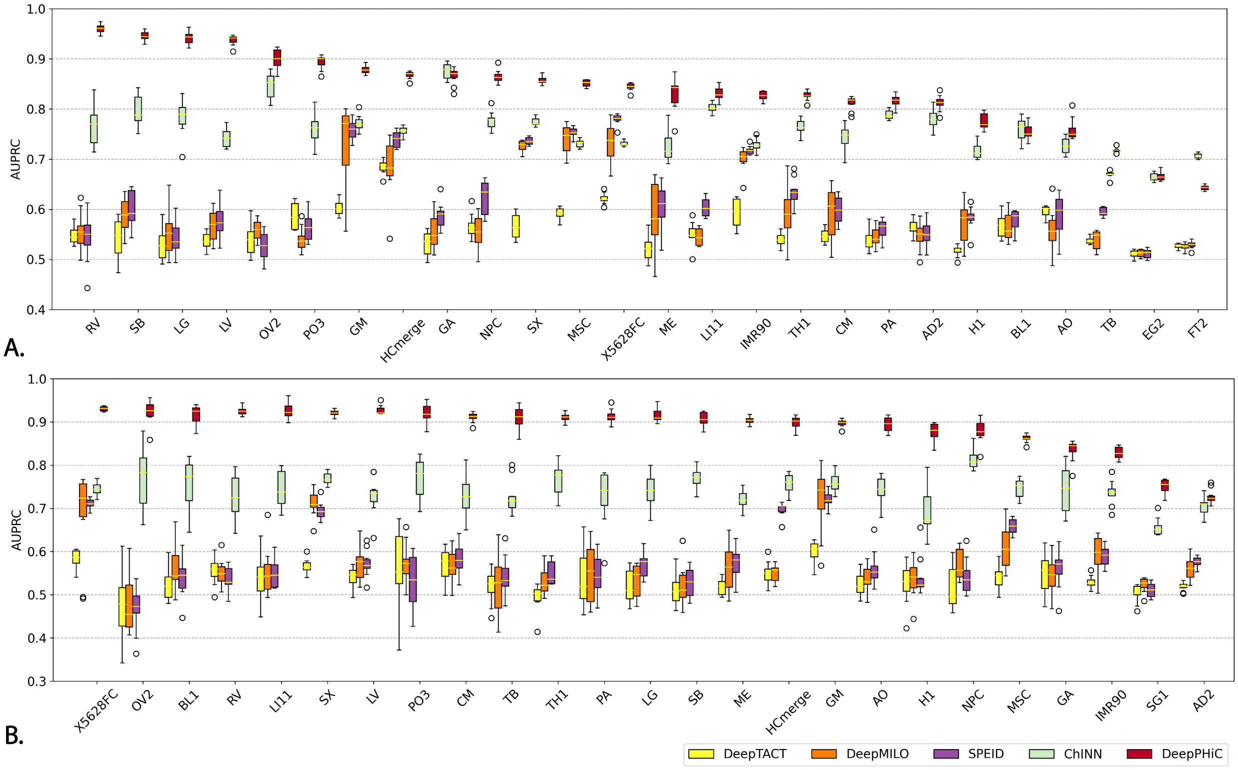


Figure S7: Comparison of DeepPHiC and state-of-the-art deep learning models on predicting **A.** Promoter-enhancer interactions (PE) and **B.** Promoter-promoter interactions (PP). Each boxplot represents the AUPRC in 10 experiments for each tissue. FDR cutoffs for constructing both positive set and negative set are FDR<0.1 and FDR>0.7.

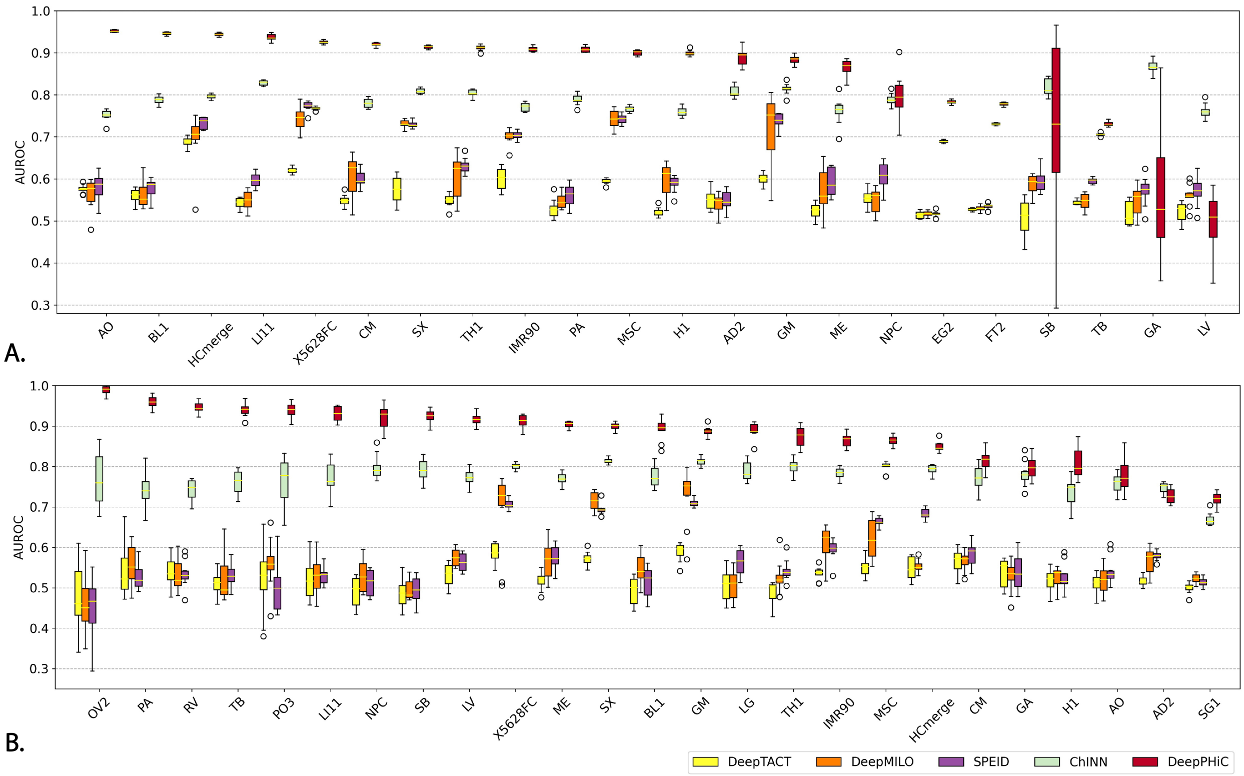


Figure S8: Comparison of DeepPHiC and state-of-the-art deep learning models on predicting **A.** Promoter-enhancer interactions (PE) and **B.** Promoter-promoter interactions (PP). Each boxplot represents the AUC in 10 experiments for each tissue. FDR cutoffs for constructing both positive set and negative set are FDR<0.1 and FDR>0.9.

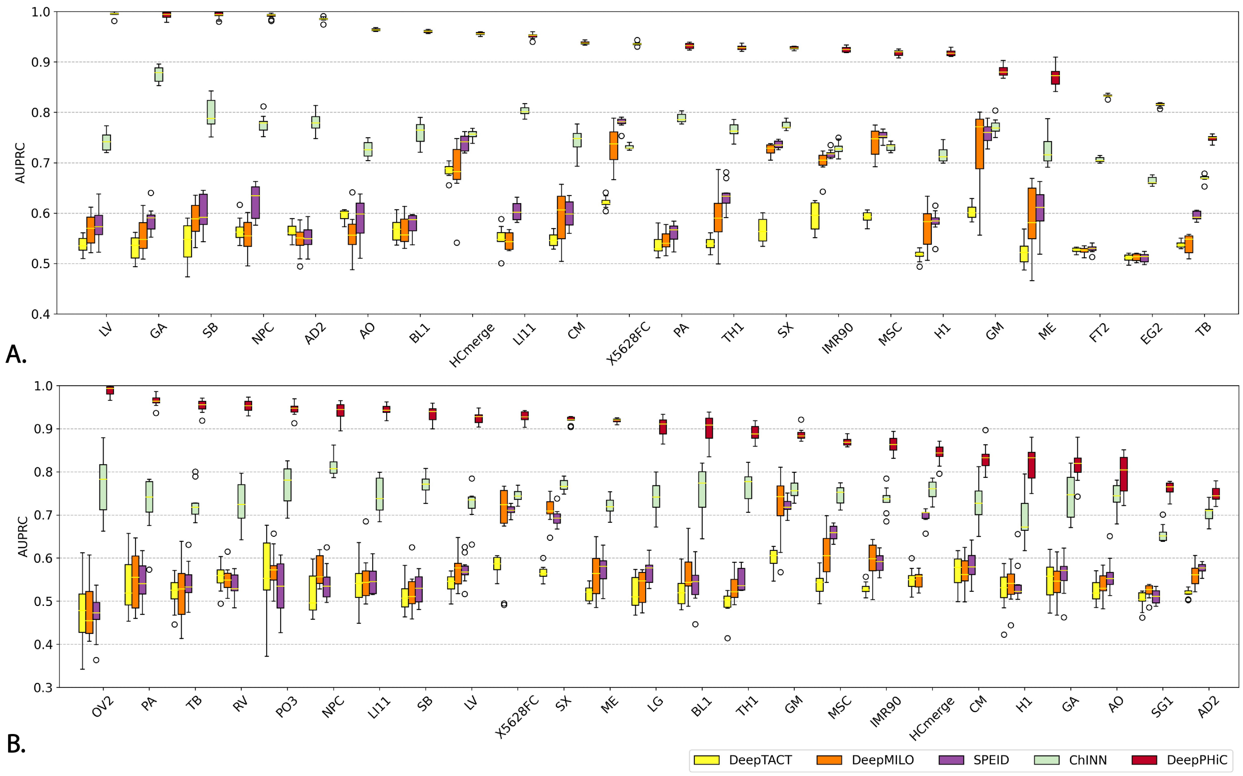


Figure S9: Comparison of DeepPHiC and state-of-the-art deep learning models on predicting **A.** Promoter-enhancer interactions (PE) and **B.** Promoter-promoter interactions (PP). Each boxplot represents the AUPRC in 10 experiments for each tissue. FDR cutoffs for constructing both positive set and negative set are FDR<0.1 and FDR>0.9.

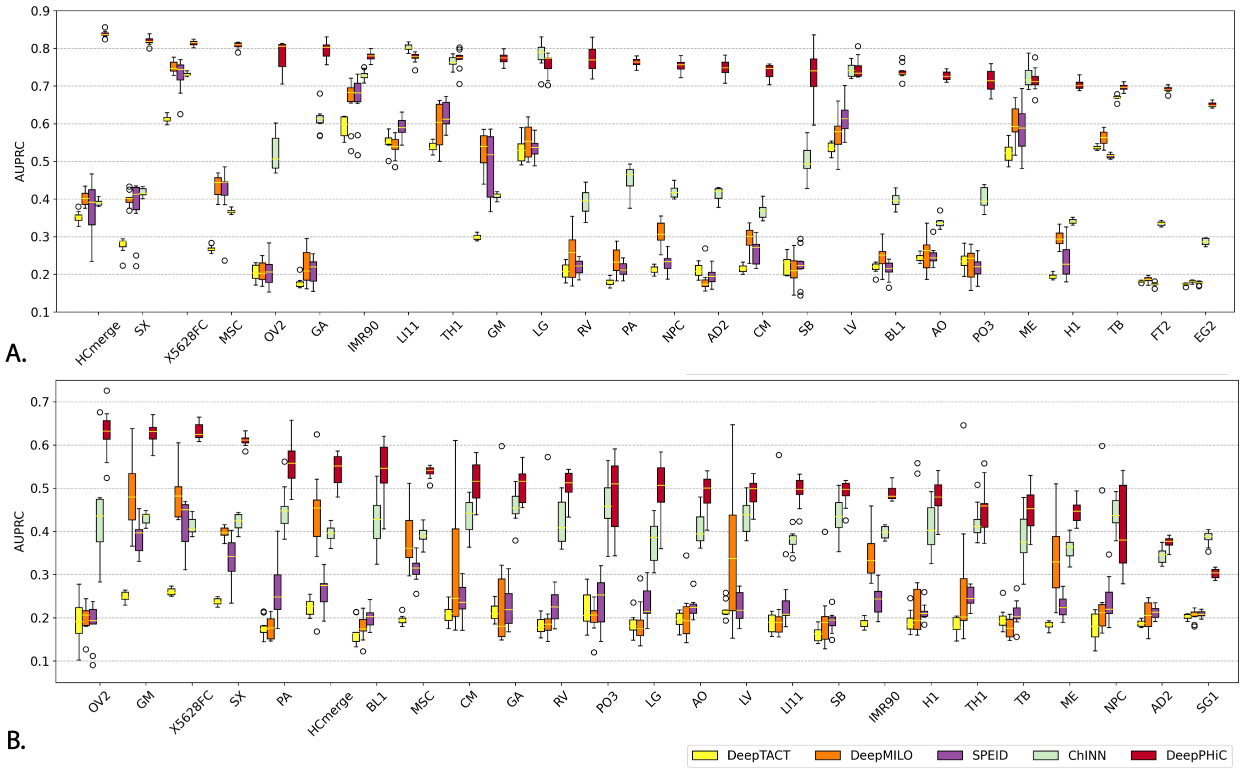


Figure S10: Comparison of DeepPHiC and state-of-the-art deep learning models on predicting **A.** Promoter-enhancer interactions (PE) and **B.** Promoter-promoter interactions (PP). Each boxplot represents the AUPRC in 10 experiments for each tissue. The number of chromatin interactions in the negative set are five times the number of chromatin interactions in the positive set.

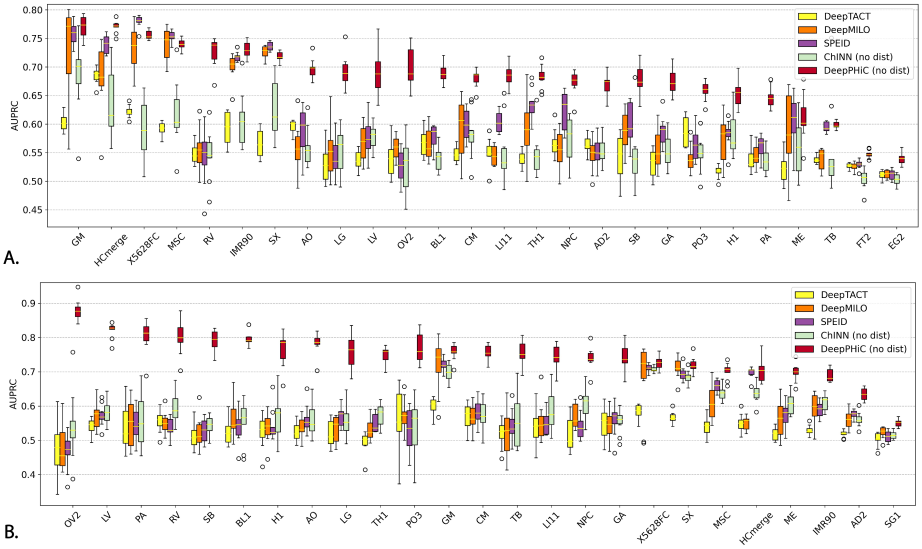


Figure S11: Compare DeepPHiC without distance (DeepPHiC (no dist))and ChINN without distance (ChINN (no dist)) to other deep learning models for predicting **A.** Promoter-enhancer interactions (PE) and **B.** Promoter-promoter interactions (PP). Each boxplot represents the AUC in 10 experiments for each tissue.

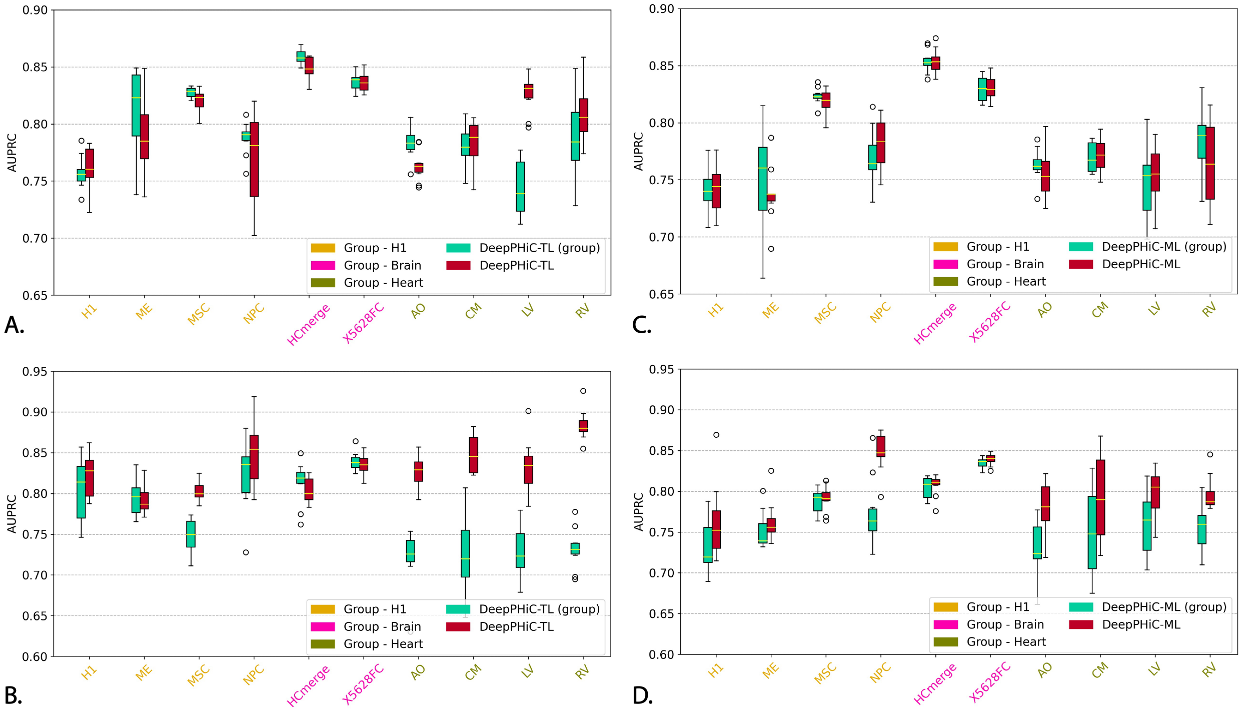


Figure S12: Compare DeepPHiC-TL and DeepPHiC-ML utilizing biologically relevant tissues/cell types and all tissues/cell types on predicting **A.** Promoter-enhancer interactions using DeepPHiC-TL and DeepPHiC-TL (group); **B.** Promoter-promoter interactions using DeepPHiC-TL and DeepPHiC-TL (group); **C.** Promoter-enhancer interactions using DeepPHiC-ML and DeepPHiC-ML (group); **D.** Promoter-promoter interactions using DeepPHiC-ML and DeepPHiC-ML (group). Each boxplot represents the AUC in 10 experiments for each tissue/cell type. DeepPHiC-ML: DeepPHiC-ML uses all tissues/cell types. DeepPHiC-ML (group): DeepPHiC-ML uses biologically relevant tissues/cell types. DeepPHiC-TL: DeepPHiC-TL uses all relevant tissues/cell types. DeepPHiC-TL (group): DeepPHiC-TL uses biologically relevant tissues/cell types.

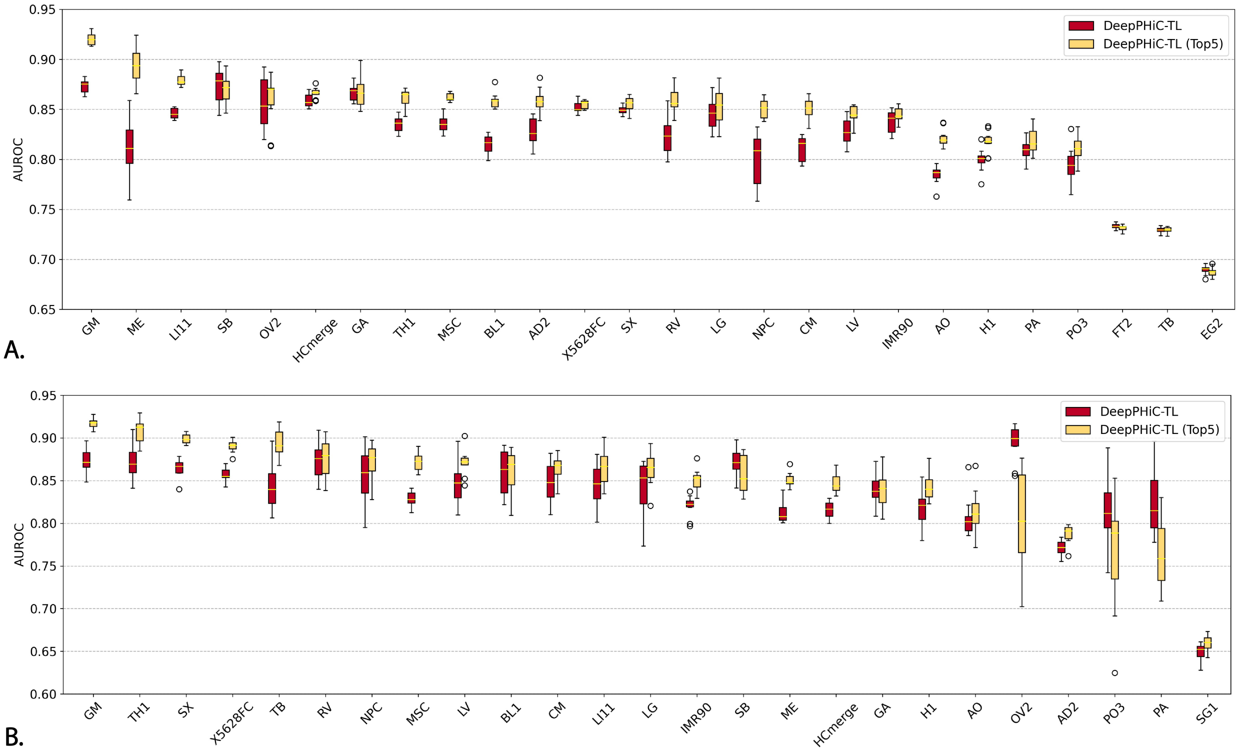


Figure S13: Compare DeepPHiC-TL(Top5) to the default setting of DeepPHiC-TL, which utilizes all other tissues/cell types. Here, we choose top five tissues/cell types of highest biological similarity to the tissue/cell type of interest for pre-training DeepPHiC-TL, which is denoted as DeepPHiC-TL (Top5). The biological similarity between tissues/cell types based on the common genes in the proximity of two anchors in chromatin interactions. **A.** Promoter-enhancer interactions (PE) and **B.** Promoter-promoter interactions (PP). Each boxplot represents the AUC in 10 experiments for each tissue.

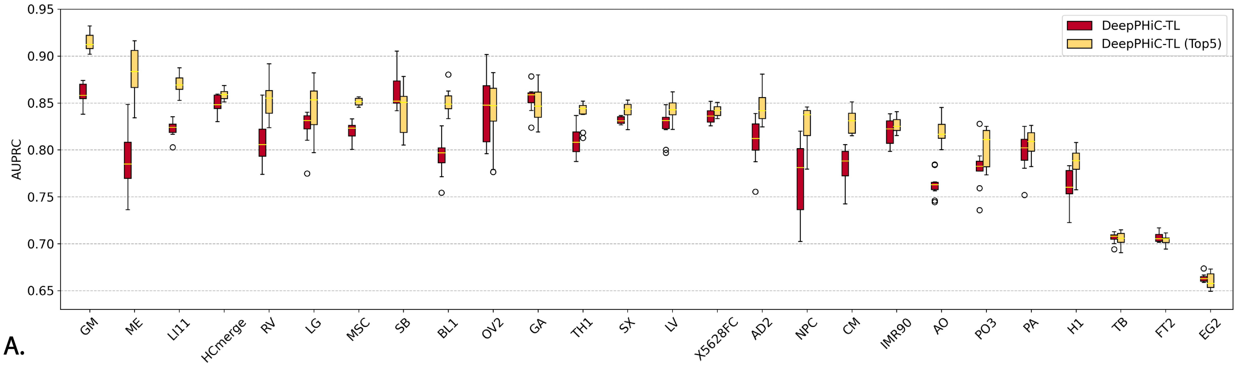


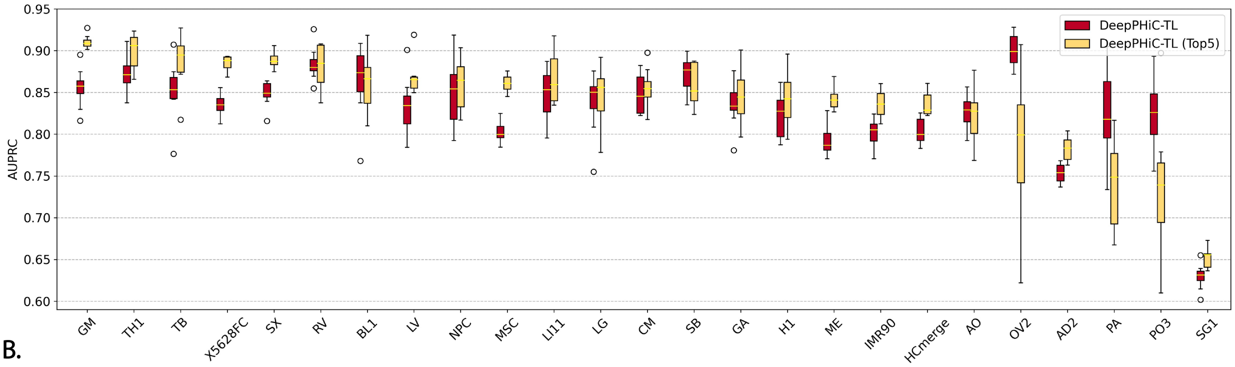


Figure S14: Compare DeepPHiC-TL(Top5) to the default setting of DeepPHiC-TL, which utilizes all other tissues/cell types. Here, we choose top five tissues/cell types of highest biological similarity to the tissue/cell type of interest for pre-training DeepPHiC-TL, which is denoted as DeepPHiC-TL (Top5). The biological similarity between tissues/cell types based on the common genes in the proximity of two anchors in chromatin interactions. **A.** Promoter-enhancer interactions (PE) and **B.** Promoter-promoter interactions (PP). Each boxplot represents the AUPRC in 10 experiments for each tissue.

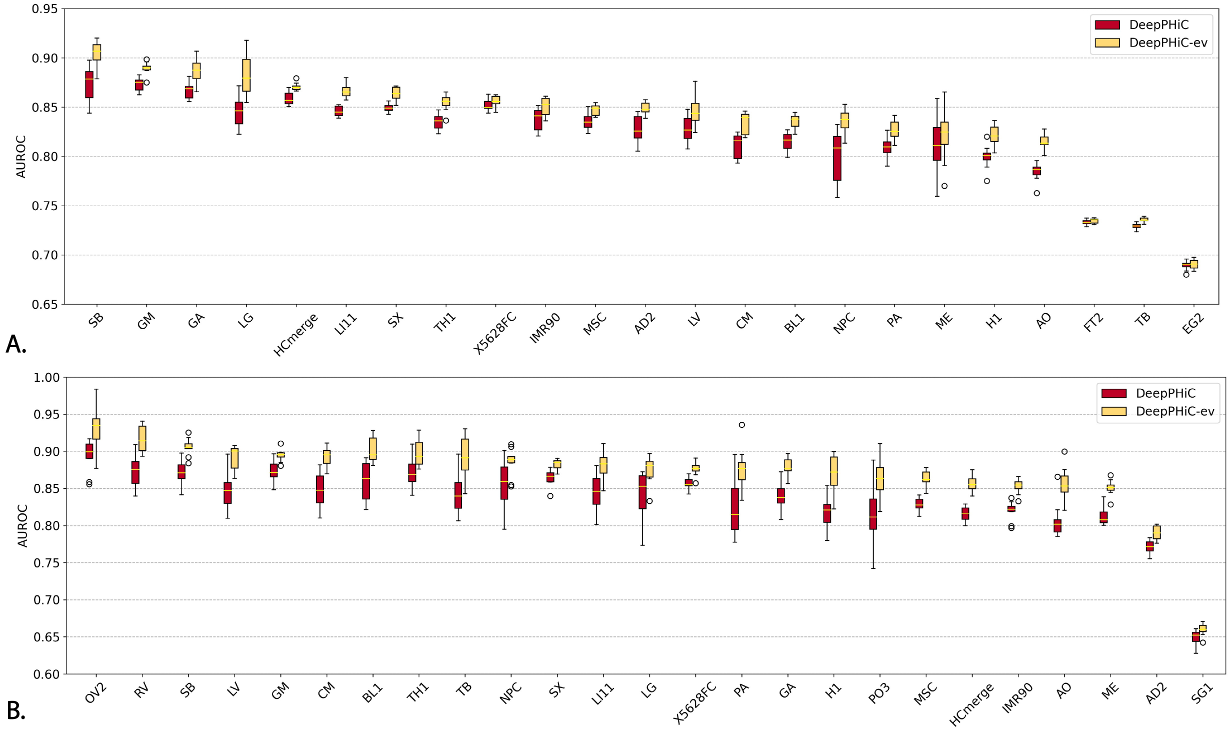


Figure S15: Compare DeepPHiC-ev to DeepPHiC in the transfer learning paradigm. DeepPHiC-ev utilizes the evolutionary information in terms of phastCons scores in the DNA sequence as an additional input feature in DeepPHiC. **A.** Promoter-enhancer interactions (PE) and **B.** Promoter-promoter interactions (PP). Each boxplot represents the AUC in 10 experiments for each tissue.

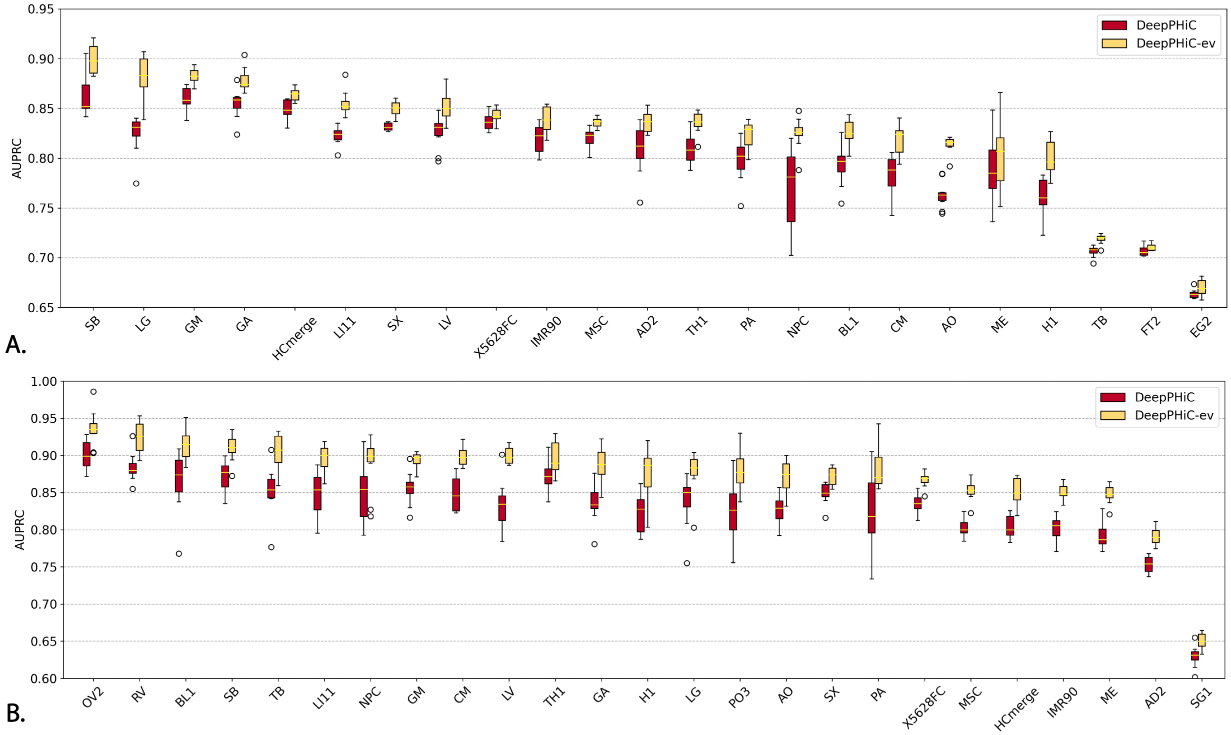


Figure S16: Compare DeepPHiC-ev to DeepPHiC in the transfer learning paradigm. DeepPHiC-ev includes the evolutionary information in terms of phastCons scores in the DNA sequence as an additional input feature in DeepPHiC. **A.** Promoter-enhancer interactions (PE) and **B.** Promoter-promoter interactions (PP). Each boxplot represents the AUPRC in 10 experiments for each tissue.

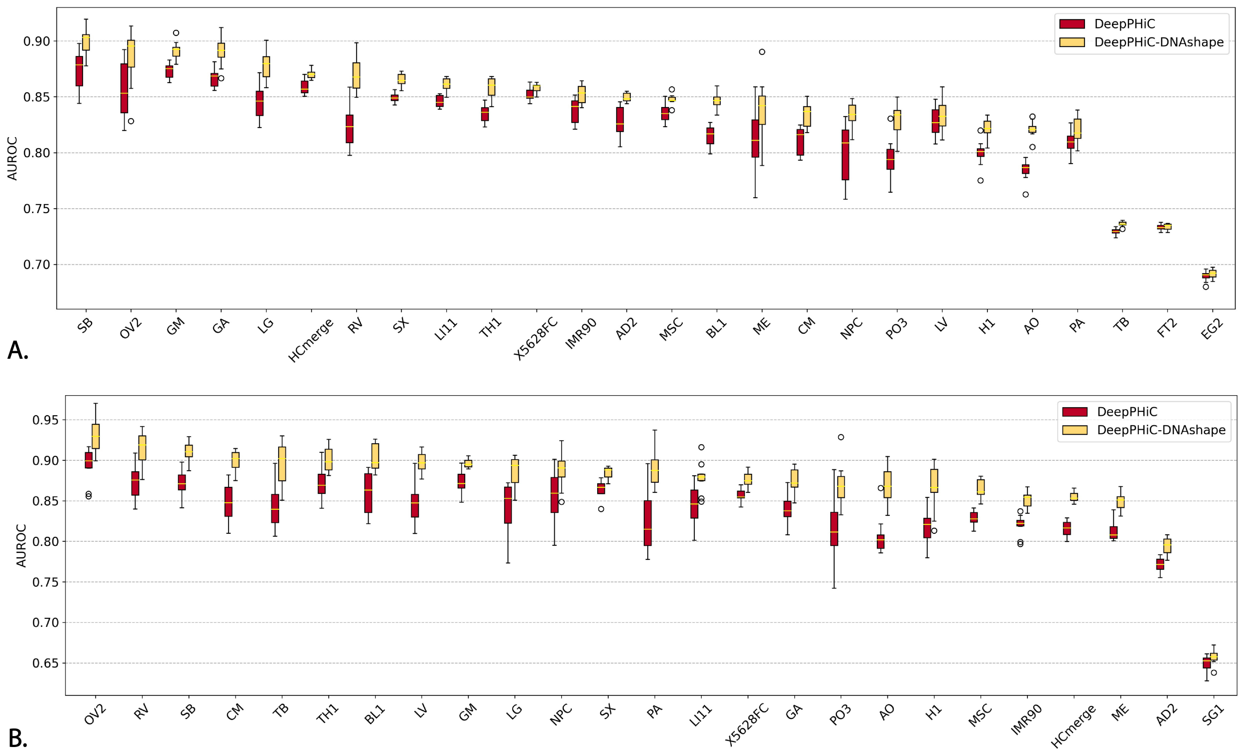


Figure S17: Compare DeepPHiC-DNAshape to DeepPHiC in the transfer learning paradigm. DeepPHiC-DNAshape includes DNA structural features such as as an additional input feature in DeepPHiC. **A.** Promoter-enhancer interactions (PE) and **B.** Promoter-promoter interactions (PP). Each boxplot represents the AUROC in 10 experiments for each tissue.

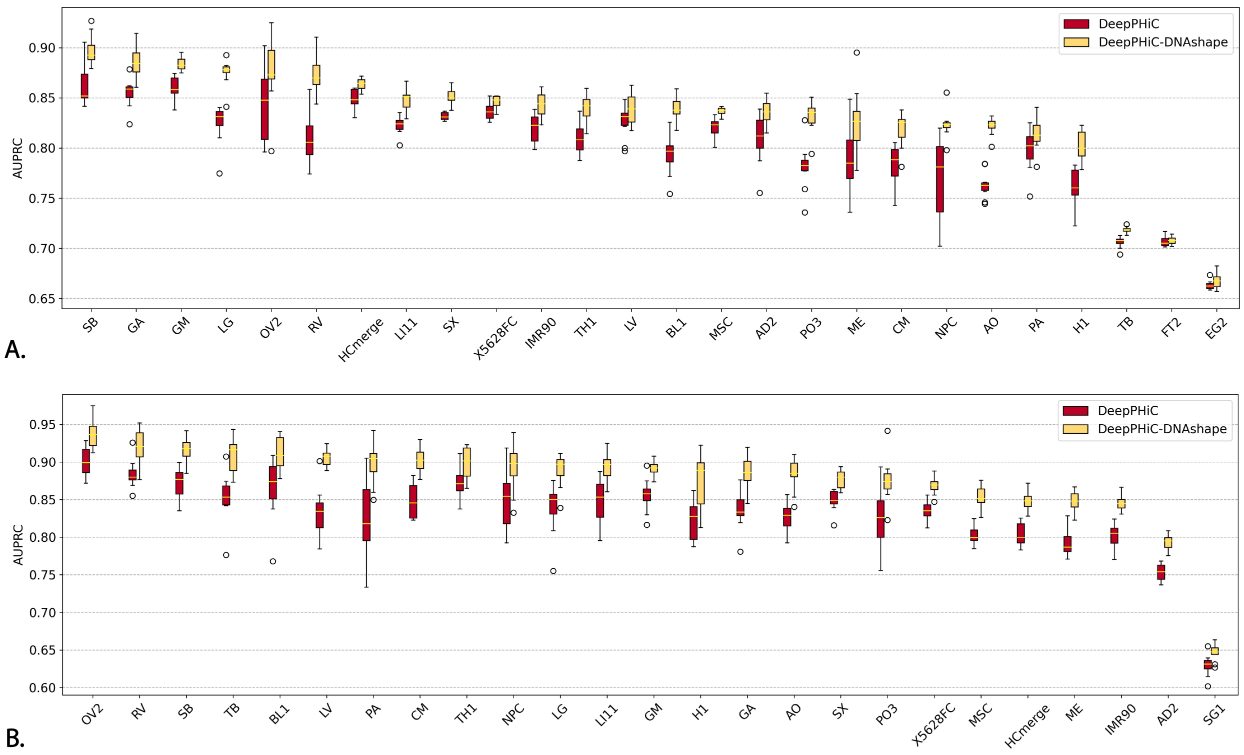


Figure S18: Compare DeepPHiC-DNAshape to DeepPHiC in the transfer learning paradigm. DeepPHiC-DNAshape includes DNA structural features such as as an additional input feature in DeepPHiC. **A.** Promoter-enhancer interactions (PE) and **B.** Promoter-promoter interactions (PP). Each boxplot represents the AUROC in 10 experiments for each tissue.
